# Energy coupling mechanisms of AcrB-like RND transporters

**DOI:** 10.1007/s41048-017-0042-y

**Published:** 2017-09-25

**Authors:** Xuejun C. Zhang, Min Liu, Lei Han

**Affiliations:** 1 National Laboratory of Biomacromolecules, CAS Center for Excellence in Biomacromolecules, Institute of Biophysics, Chinese Academy of Sciences, Beijing, 100101 China; 2 College of Life Science, University of Chinese Academy of Sciences, Beijing, 100049 China

**Keywords:** AcrB, RND transporter, Membrane potential

## Abstract

Prokaryotic AcrB-like proteins belong to a family of transporters of the RND superfamily, and as main contributing factor to multidrug resistance pose a tremendous threat to future human health. A unique feature of AcrB transporters is the presence of two separate domains responsible for carrying substrate and generating energy. Significant progress has been made in elucidating the three-dimensional structures of the homo-trimer complexes of AcrB-like transporters, and a three-step functional rotation was identified for this class of transporters. However, the detailed mechanisms for the transduction of the substrate binding signal, as well as the energy coupling processes between the functionally distinct domains remain to be established. Here, we propose a model for the interdomain communication in AcrB that explains how the substrate binding signal from the substrate-carrier domain triggers protonation in the transmembrane domain. Our model further provides a plausible mechanism that explains how protonation induces conformational changes in the substrate-carrier domain. We summarize the thermodynamic principles that govern the functional cycle of the AcrB trimer complex.

**Electronic supplementary material:**

The online version of this article (doi:10.1007/s41048-017-0042-y) contains supplementary material, which is available to authorized users.

## Introduction

In Gram-negative bacteria, the periplasm functions as a protective buffer zone against cytotoxic substances from the extracellular environment (Li* et al*. 2015). The cell actively expels toxic compounds that have penetrated the outer membrane and that have accumulated in the periplasmic space, thus preventing the toxins from either harming the cell from within the periplasm, or further penetrating into the cytosol. In addition, metabolic waste and other cytotoxic compounds transported from cytosol to periplasm should also be expelled promptly. Resistance-nodulation-cell division (RND) type of transporters plays essential roles in these toxin-extrusion processes vital for cell survival (Higgins 2007).

RND transporters form a superfamily of membrane proteins that play crucial roles in diverse biological processes from multidrug resistance in bacteria to trafficking of lipid molecules in eukaryotic cells (Higgins 2007). RND proteins unidirectionally transport substrates between two distinct micro-environments not necessarily separated by a biological membrane. In particular, RND-mediated transport often occurs against the substrate chemical potential,* e.g*., from a low-concentration to a high-concentration environment, or from a high-affinity carrier to a low-affinity carrier. Such transport requires external energy, and RND transporters are known to utilize the electrochemical potential of protons,* i.e*., the proton motive force (PMF), to drive their substrate-transport processes (Yamaguchi* et al*. 2015).

The AcrAB–TolC tripartite complex, a prototypical member of the RND family, is constitutively expressed in many Gram-negative bacteria, is responsible for efflux transport of lipophilic and/or amphipathic compounds (a common property of many cytotoxic substances such as antibiotics), and thus plays important roles in multidrug resistance (Nikaido and Takatsuka 2009; Yamaguchi* et al*. 2015). In this complex, AcrB captures a wide variety of structurally dissimilar drugs and toxic compounds from either periplasm or the periplasmic leaflet of the inner membrane and delivers them to a conduit formed by a TolC trimer which connects to the exterior of the cell. Analysis of amino acid sequences shows that AcrB belongs to the Hydrophobe/Amphiphile Efflux-1 (HAE1) family of the RND superfamily (Perrin* et al*. 2010). Hereafter, we refer to members of the HAE1 family and other phylogenetically related RND families as AcrB-like transporters (Fig. S1). From the view of energy coupling, AcrB-like transporters are distinct from ATP-binding cassette (ABC) transporters. In most cases, ABC transporters utilize cytosolic energy source (*i.e*., ATP hydrolysis) to drive cross-membrane transport of substrates. In contrast, AcrB-like transporters utilize PMF across the bacterial inner membrane to transport substrates, either within an aqueous environment or at the aqueous–membrane interface.

Structures, substrate recognition mechanisms, and the functional cycle of AcrB-like transporters have been extensively reviewed previously (Higgins 2007; Nikaido and Takatsuka 2009; Li* et al*. 2015; Yamaguchi* et al*. 2015). Here, we focus on the structural basis of the energy coupling mechanisms of AcrB-like transporters. We will first summarize the structural features of the AcrB complex for the sake of discussion of the mechanistic principles, and then propose a model on the energy coupling mechanism of this family of transporters between the substrate-carrier domain and the transmembrane (TM) PMF-driven motor domain. All arguments discussed below are based on insights from *Escherichia coli* AcrB (Ec-AcrB), unless stated otherwise.

## Basic Structures of AcrB

**Fig. 1:**
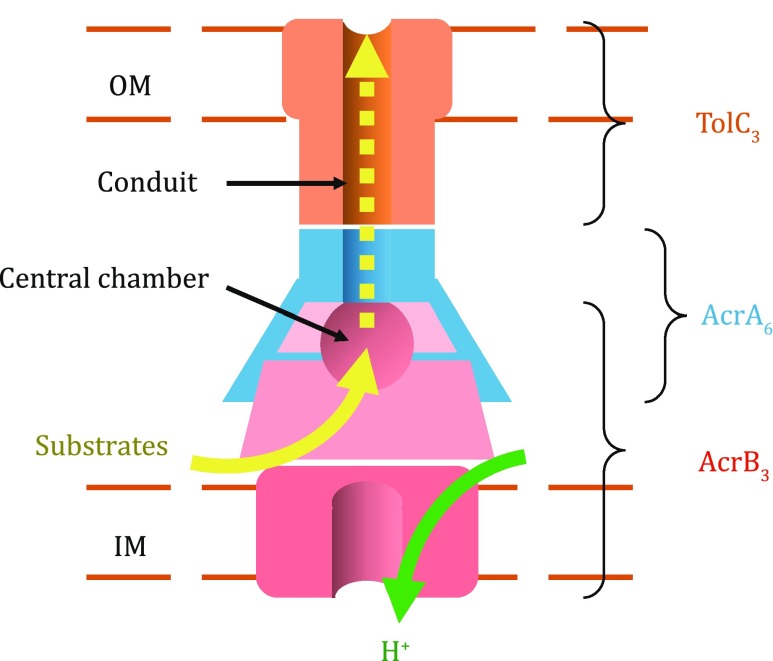
Schematic diagram of the AcrAB–TolC complex. “OM” and “IM” stand for outer and inner membranes, respectively

The AcrAB–TolC tripartite complex from *E. coli* is the most extensively studied RND transporter (Yamaguchi* et al*. 2015). Recent cryo-electron microscopy studies revealed that this complex is of a stoichiometry of AcrB_3_:AcrA_6_:TolC_3_ (Du* et al*. 2014; Jeong* et al*. 2016). As shown schematically in Fig. 1, AcrB serves as the PMF-driven transporter and forms a homo-trimer; the TolC trimer forms a conduit connecting the exit chamber of the AcrB trimer to exterior of the cell; and the AcrA hexamer serves as an adaptor between the AcrB and TolC trimers.

**Fig. 2:**
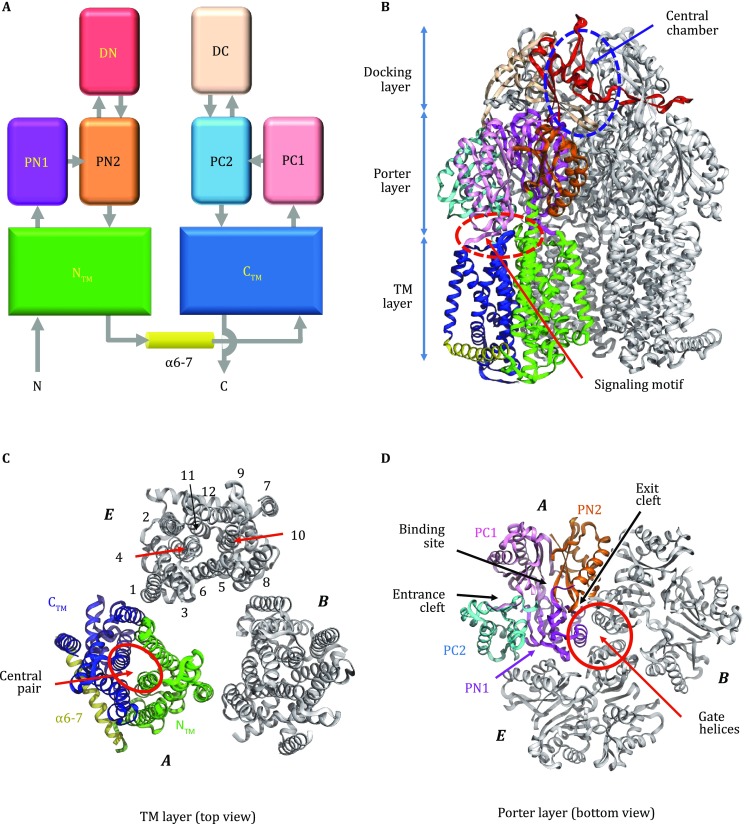
Crystal structures of the AcrB asymmetric trimer. **A** The topology of the AcrB protomer. **B**–**D** The AcrB trimer (PDB ID: 4DX5) is shown in ribbon diagrams. In each panel, the access protomer is shown in color, while the other two protomers are displayed in gray. In **C** and **D**, the access, binding, and extrusion protomers are labeled as “*A*”, “*B*”, and “*E*,” respectively. In the top view of the TM layer (**C**), TM helices are labeled as “1” through “12”

**Fig. 3:**
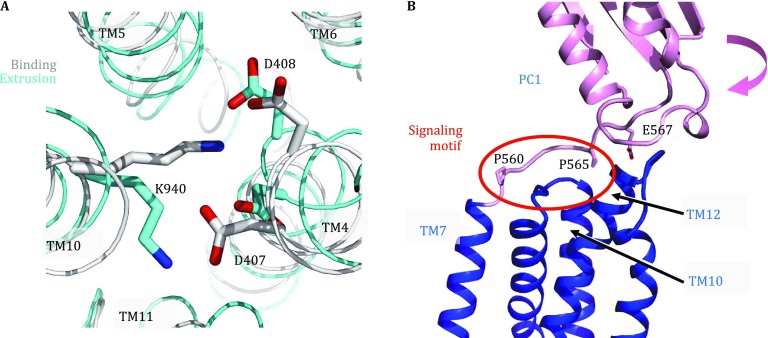
Protonation switch in the TM domain. **A** The D407-D408-K940 cluster, viewed from the periplasm direction. The TM domains of the binding (gray) and extrusion (cyan) protomers are superimposed, with an overall RMSD of 1.3 Å for 314 Cα-atom pairs. **B** Side view of the signaling motif between C_TM_ (blue) and PC1 (pink). The substrate binding induced clockwise rotation of PC1 is postulated to further induce a clockwise rotation of TM10, which triggers protonation in TM4

Among the three component proteins in the *E. coli* AcrAB–TolC complex, AcrB (GenBank access ID: ANK05811.1), consisting of 1,049 amino acid residues, is the most extensively studied protein both structurally and functionally. Currently, the Protein Data Bank (PDB) database (Berman* et al*. 2000) contains over 40 full-length AcrB crystal structures, demonstrating that the stable homo-trimer is the functional form of AcrB. Each protomer is composed of three major domains, namely the TM domain; the substrate-carrier, porter domain (formerly called port domain); and the TolC-docking domain (Murakami* et al*. 2002) (Fig. 2). The TM domain contains 12 TM helices (TMs 1–12) (Fig. 2C; Fig. S1A) and resides in the inner membrane with both its N- and C-termini located in the cytosol. This domain assumes a pseudo twofold symmetry between the N-terminal subdomain (termed N_TM_, composed of TMs 1–6) and C-terminal subdomain (C_TM_, TMs 7–12), with the rotation axis perpendicular to the membrane. Around the symmetry axis, TM4 and TM10 form a parallel central helix-pair. Functionally important residues D407, D408, and K940 (numbered as in Ec-AcrB) are located in the TM4–TM10 interface (Fig. 3A) and form a rather extensive hydrogen (H)-bond network (Middlemiss and Poole 2004; Eicher* et al*. 2012). In particular, D407 is located in a highly conserved sequence motif, GX_3_DX_6_E, of AcrB-like transporters (Perrin* et al*. 2010) (Fig. S1). Integrity of this H-bond network is essential for the transport function of AcrB-like transporters, and the D407–D408 acidic pair is identified to be the protonation site required for PMF-energy conversion (Murakami* et al*. 2002). Furthermore, between N_TM_ and C_TM_ (particularly between TMs 6 and 7), a long amphipathic helix (~26 residues, termed α6-7) represents a conserved structural feature of RND transporters (Fig. 2) (Murakami* et al*. 2002; Sennhauser* et al*. 2009; Gong* et al*. 2016). It presumably lies on the cytosolic surface of the inner membrane, forming part of the rim of the AcrB trimer complex. A highly conserved tyrosine residue, Y527 (Fig. S1), anchors α6-7 to another conserved region at the C-terminal end of TM12.

The porter domain contains four topologically homologous subdomains, namely PN1, PN2, PC1, and PC2, each of which consists of two β-α-β repeats (Fig. 2). The four β-strands in each subdomain form a β-sheet which participates in the formation of the substrate-transport path. In the primary sequence, PN1 and PN2 are tandemly inserted between TMs 1 and 2 of N_TM_, and PC1 and PC2 between TMs 7 and 8 of C_TM_ (Fig. 2A; Fig. S1A). Furthermore, in the 3D structure, PN1 and PC2 pack together via a shared β-sheet, forming a rigid body (Eicher* et al*. 2014). So do PN2 and PC1, albeit significantly more flexible (when comparing different protomers). Thus, the porter domain in each AcrB protomer may be considered to be composed of two structural units, termed PN1-PC2 and PN2-PC1. The PN2-PC1 unit is observed to possess one of the major substrate binding sites (referred to as distal/deep binding site) (Murakami* et al*. 2006). The substrate entrance (also called proximal binding site) is located between the subdomains PC1 and PC2, laterally facing the periplasmic space. The substrate exit is located between the subdomains PN1 and PN2, connecting to a central exit chamber of the AcrB trimer. Thus, the entire substrate-transport path (including the entrance cleft, “major” binding site, and exit cleft) is formed between the two units, PN1-PC2 and PN2-PC1 (Li* et al*. 2015) (Fig. 2D). Importantly, exchanging the porter domains of AcrB and AcrD (an AcrB homolog) changes substrate specificity of the corresponding chimeric transporters (Elkins and Nikaido 2002). This result and other related studies firmly establish that the porter domain is the substrate-carrier domain of AcrB and determines the substrate specificity. In addition, the docking domain of an AcrB protomer is composed of two subdomains of similar folding, termed as DN (protruded from PN2) and DC (protruded from PC2), each of which also consists of two β-α-β repeats. Taken together, at the level of folding topology, both the porter domain and the docking domain of an AcrB protomer possess an internal pseudo twofold symmetry, which is an extension of the twofold symmetry of the TM domain (Fig. 2A).

In the homo-trimer, AcrB molecules form three layers parallel to the membrane plane (Fig. 2): (1) The three TM domains form a loosely packed ring structure located in the inner membrane. (2) The three porter domains form a tightly packed, membrane-proximal layer in the periplasm. A three-helix bundle is formed around the pseudo threefold-symmetry axis, with each protomer contributing one helix (termed gate helix, formerly called pore helix) from its PN1 subdomain. (3) The docking domains interlock with each other using a protruding β-hairpin (known as “peg”) from each of the DN and DC subdomains, forming a rigid, threefold symmetrical, membrane distal layer. Folding homology between DN and DC subdomains further renders the layer an approximate sixfold symmetrical architecture, which may facilitate interactions of the docking layer with the homo-hexamer of the adapter AcrA in the assembled complex (Jeong* et al*. 2016). Furthermore, the docking-domain trimer forms the central exit chamber (formerly called funnel), which extends into the cellular exterior via the TolC conduit (Fig. 1).

## Peristaltic Mechanism of AcrB

Based on asymmetric tripartite structures of AcrB reported from multiple laboratories (Murakami* et al*. 2006; Seeger* et al*. 2006; Sennhauser* et al*. 2007), a peristaltic mechanism containing a three-step functional rotation has been proposed. In this functional cycle, each AcrB protomer successively runs through the access (A), binding (B), and extrusion (E) phases (also known as loose, tight, and open states). Furthermore, in a given crystal structure of the asymmetric trimer of AcrB, each protomer assumes one of the three different phases, with a counter-clockwise phase rotation (viewed from the TolC direction). Thus, although containing no physically rotating parts, the periplasmic porter domain cycles through three sequential phases, (1) capturing a lipophilic substrate from the peripheral periplasm–membrane interface, (2) transferring the substrate to the deep binding pocket, and (3) squeezing the substrate through the exit cleft into the central chamber.

In the functional cycle, the subdomains PN1, PN2, PC1, and PC2 change their packing conformations (Eicher* et al*. 2014). First, the entrance cleft between PC1 and PC2 opens laterally to the periplasm in the access phase, but gets partially closed in the binding phase. It is completely closed in the extrusion phase, with a relative rotation of 25° between PC1 and PC2 during the binding-to-extrusion (B-E) transition. Secondly, the “major” binding site between PN2 and PC1 is of a more extended conformation in the access and binding phases, but contracts in the extrusion phase (with a relative rotation of 21°) essentially eliminating the substrate binding. Thirdly, the exit cleft between PN1 and PN2 is closed in the binding phase, but becomes open to the central chamber in the extrusion phase. This opening is partially caused by an 18° relative rotation between PN1 and PN2 during the B-E transition, and partially by a gating movement of the gate helix from a neighboring promoter that is under the extrusion-to-access (E-A) transition.

This functional cycle requires external energy to drive, which is provided by the PMF-driven motors located in the three TM domains, in a fashion analogous to a three-cylinder car engine. As substrate transport and power generation are two functions physically separated (in the porter and TM domains, respectively), reliable communication and energy coupling between the two corresponding layers are essential for ensuring proper energy-transduction efficiency and thus high transport rate. Questions remaining to be addressed concerning the peristaltic mechanism include “How does substrate binding in the porter domain activate protonation of the TM domain?” and “How does protonation in the TM domain in turn drive the conformational change in the porter domain?” Finding answers to these questions will allow for a better understanding of the mechanistic details of RND transporters.

## Protonation in AcrB Transporter

As pointed out earlier, the protonation site in each Ec-AcrB protomer is composed of residues D407, D408 in TM4, and K940 in TM10. The asymmetric trimer structure of AcrB provides a number of clues as to how this putative protonation site may change its local conformation between distinct phases. The D407–D408 pair is located in the middle of the lipid bilayer and is insulated from bulk solvent. Because of the low dielectric constant of their environment, either of these two acidic residues may potentially be protonated. (See Ref. (Hanz* et al*. 2016) for an NMR study on the protonation of acidic residues in a TM helix.) On the other hand, by forming salt-bridge bonds, the nearby basic residue K940 may reduce the protonation ability (*i.e*., p*K*_a_) of the acidic pair. In both the access and binding protomers, the positive charge of the K940 side chain is located between the two negatively charged side chains of D407 and D408, preventing the acid residues from being protonated. The equilibrium conformation of such a deprotonated state (including its relative position to the membrane) will be considered as a reference for conformational changes upon protonation (see below). In contrast, in the extrusion protomer, the lysine side chain moves away from the acidic residues (Fig. 3A) and is stabilized by a H-bond formed with another functionally important residue T978 in TM11 (Takatsuka and Nikaido 2006). Therefore, in the extrusion phase p*K*_a_ values of both aspartate residues increase, so that one of them (presumably of higher p*K*_a_) will become protonated (Li* et al*. 2015). Once one of the two residues is protonated, the p*K*_a_ of the other residue will decrease. Thus, it is unlikely that these two acidic residues are protonated simultaneously.

Next, we will discuss the sources and sinks of proton flux. Based on available structural evidence we put forward the following argument: Upon activation of the TM domain, a proton (carrying a positive charge) moves from the periplasmic side of the inner membrane, following the negative-inside electrostatic membrane potential (∆*Ψ*), to the proton binding site at the D407–D408 pair. As illustrated in the 1.9 Å-resolution crystal structure of AcrB (PDB ID: 4DX5), the proton-transfer wire, through which protons move, is likely to be formed by side chains of polar residues in TM helices as well as by membrane-embedded water molecules (Eicher* et al*. 2012). For examples, the highly conserved residues P906, G930, N941, R971, and G1010 (Fig. S1) are either directly involved in the formation of the proton-transfer wires, or facilitate binding of water molecules. Thus, the proton is likely to be transferred from a periplasmic pool to the D407–D408 pair via a mechanism similar to the Newton’s cradle. Moreover, once the protomer passes the E-A transition state, the positive charge of the K940 side chain returns to the vicinity of the D470–D408 pair, and deprotonation occurs. The released proton will continue its cross-membrane journey, presumably releasing the rest of its PMF energy. As part of the proton release path, conserved R971 in TM11 is located on the cytosolic side of the D407–D408 pair. In particular, in the access and binding phases D407 is connected to R971 via a cluster of four water molecules, while in the extrusion phase this proton path is blocked by the two side chains of V411 and L944 which are conserved as hydrophobic residues in the AcrB-like family (Fig. S1). Taken together, depending on the protonation status, two distinct constellations of H-bond networks are found inside the TM domain, thus establishing the pathway for proton loading and release.

A similar mechanism of substrate binding-induced protonation has been proposed for some transporters of the major facilitator superfamily (MFS) (Zhao* et al*. 2014). In the bacterial proton-dependent oligopeptide transporters (POTs), which import di- or tri-peptides by utilizing PMF energy, a cluster of conserved polar residues is present in the middle of the transmembrane region nearby the substrate binding site. This cluster typically includes one (or two) acidic residue(s), one (or two) positively charge residue(s), and other uncharged polar residues. For example, in the *E. coli* peptide-H^+^ symporter, YbgH, residues Q18, E21, Y22, and K118 form such a cluster. Upon binding a substrate peptide, the negatively charged carboxyl terminal group of the substrate attracts the positively charged side chain of K118 away from the clusters, thus promoting protonation of E21. Once E21 becomes protonated, ∆*Ψ* exerts an inward force to the transporter, triggering the outward-to-inward conformational change of the symporter. Regarding AcrB-like transporters, D408 is less conserved than D407 (Fig. S1A). Thus, many members from this transporter family have only one acidic residue as the protonation site. As suggested in the above YbgH case, a single acidic residue may function effectively as a protonation site, provided that some electro-negative groups are present in its vicinity. In addition, as shown in Fig. S1, the presence of a basic residue in TM10 is not absolutely essential for alternating the protonation status of TM4. Nevertheless, removing a positive charge (*e.g.*, K940 in Ec-AcrB) is likely accompanied by eliminating a negative charge (*e.g*., D408), and thus the protonation ability of the polar cluster is maintained. In such a case, the p*K*_a_ value of the remaining buried acidic residue might be reduced by being approached by an H-bond donor. A similar deprotonation mechanism has been proposed recently for activation of class-A GPCRs (Zhang* et al*. 2014).

Results from several previous studies support our hypothesis. For instance, the D407-D408-K940 cluster of MexB (an AcrB homolog) has been shown to be very sensitive to point mutations. Any mutation that causes a charge change (*e.g*., D407N and D408N) results in loss of transport activity (Guan and Nakae 2001; Middlemiss and Poole 2004). In addition, a chemical modification assay using DCCD (dicyclohexylcarbodiimide) as a probe, which identifies protonatible acidic residue(s) in a hydrophobic environment, showed that D408 of Ec-AcrB can be protonated (Seeger* et al*. 2009). Recently, mutations in the proposed proton release path (residues V411, L944, and R971) have been shown to be lethal (Liu and Zhang 2017). These results support the notion that the D407–D408 pair forms part of the proton-transfer wire for the PMF-driven mechanism.

## Energy Coupling in AcrB

For Ec-AcrB, the relative movement K940 in TM10 away from the D407–D408 acidic pair in TM4 seems to represent the trigger for the protonation event occurring within the TM domain. In order to induce such a relative movement between TMs 4 and 10, the substrate binding signal must be transduced from the porter layer to the TM layer. The communication between the two layers is speculated to be long-distance in nature (Eicher* et al*. 2014; Yamaguchi* et al*. 2015), and possible mechanisms of signaling through direct physical transduction are currently ignored. By inspiring available crystal structures of AcrB, however, we proposed an alternative, interdomain contact-mediated mechanism for the signaling. We noticed that a short β-strand located inside an S-shaped loop connecting TM9 and TM10 forms a three H-bond, irregular, parallel β-sheet with another short β-strand in the loop connecting C_TM_ to PC1 (Fig. 3B). We proposed that this β-sheet provides an information path for the substrate binding signal transduced from the porter layer to the TM layer, and therefore we term this sheet PC1-C_TM_ signaling motif. Based on previously reported structures *(*Murakami* et al*. 2006; Seeger* et al*. 2006; Sennhauser* et al*. 2007), we proposed that this motif allows movement of PC1 upon substrate binding to be sensed by TM10, inducing a ~12° rotation of TM10 relative to the remaining part of C_TM_ during the B-E transition. Furthermore, during the B-E transition, a similar though smaller movement is also observed for TM4. In particular, a short β-strand connecting TM3 and TM4 forms three H-bonds with a β-strand inside the N_TM_-PN1 connecting loop, and we term this parallel β-sheet PN1-N_TM_ signaling motif. Because of the presence of this motif, a movement of PN1 upon substrate binding may be sensed by TM4. Comparing the binding and extrusion phases reveals a ~8° rotation of the N-terminal (periplasmic) half of TM4 relative to the C-terminal (cytosolic) half, resulting in a helix bulge near the conserved G403 (of the GX_3_DX_6_E motif) in the extrusion phase. As a consequence of this deformation, the side chain of D407 interacts with the backbone carbonyl oxygen of G403, presumably increasing the p*K*_a_ value of D407. Interestingly, both the short β-strands in the N_TM_-PN1 and C_TM_-PC1 loops are flanked by two conserved proline residues (Fig. 3B; Fig. S1A). Together, the relative movement (with a net 14° rotation) between TM10 and the C-terminal half of TM4 likely triggers protonation of the D407–D408 pair, using the abovementioned mechanism. Recently, it has been shown that mutations in the signaling motifs indeed reduce or abolish AcrB activity (Liu and Zhang 2017). In addition, although not directly located in the signaling motifs, several random point mutations in the porter–TM interface region were found to reduce the transport activity of MexB (*e.g*., S462F in the loop connecting TMs 5 and 6, E864K, V928M, and G1002D in the N-terminal (periplasmic) regions of TMs 8, 10, and 12, respectively) (Middlemiss and Poole 2004). More importantly, PC1 is involved in substrate binding in both the access and binding phases. During the B-E transition, PC1 exhibits the largest movement, compared to other subdomains in the porter layer (Seeger* et al*. 2006). An AcrB inhibitor that binds between PN2 and PC1 in the B-state seems to block this movement of PC1 (Nakashima* et al*. 2013), thus breaking the communication between the porter and TM layers. Taken together, we proposed that the signaling motifs, especially that of PC1-C_TM_, are essential for transducing substrate binding signal from the porter layer to TM layer.

Effective proton transfer across the membrane does not necessarily translate into efficient energy usage. For example, slippage in energy coupling might result in futile energy dissipation in theoretical models (Hill 1989). Thus, for an effective AcrB-like transporter, a mechanism of transducing the PMF energy into mechanical rearrangements in the porter layer likely exists. Similar to the case of PMF-driven MSF transporters (Zhang* et al*. 2015), the protonation at D407–D408 pair is likely to exert an extra inward force to the N_TM_-subdomain relative to the abovementioned reference state, causing a movement of the N_TM_-subdomain towards the cytosolic direction. The strength of the electrostatic force is of an order of 5 pN, if the electrostatic field of the membrane potential is uniformly distributed across the 30-Å thickness of the membrane (Zhang* et al*. 2015). A force of such a magnitude is sufficiently large to conduct many molecular biology events, such as moving of a motor protein along microtubules, separating of a dsDNA helix, or packaging a DNA molecule into a phage shell (Schnitzer* et al*. 2000; Cocco* et al*. 2001; Liu* et al*. 2014). Furthermore, embedding a proton-transfer wire into the membrane is equivalent to applying a focused membrane potential across the membrane, and thus the effective membrane thickness is reduced. Therefore, the extra electrostatic force upon protonation may be significantly larger than 5 pN. This extra electrostatic force may further be transduced from the TM domain to the porter domain via routes additional to the signaling motif,* e.g*., the structural connection between TM2 and PN2. Consequently, the corresponding subdomains in the porter domain are likely to move also in an inward direction. Such a movement will result in further conformational rearrangement necessary for the B-E transition, including a tilting of the gate helix in PN1 to block the exit cleft of the neighboring porter domain (Seeger* et al*. 2006).

As the TM domain moves out of the lipid bilayer, the hydrophobic mismatch between them will dramatically increase. This hydrophobic mismatch generates forces that partially balance the electrostatic force, such that no further out-membrane movement would be possible. Upon deprotonation, the energy stored in the form of a hydrophobic mismatch is released, returning the TM domain to its “reference” status (*i.e*., the access phase). In addition, the amphipathic helix α6-7, which is strategically located in the peripheral of the C_TM_-subdomain, keeps C_TM_ aligned with the membrane surface upon movement of the N_TM_-subdomain towards the cytosol. Thus, α6-7 serves as a pivot point and may facilitate tilting of the TM domain as a whole. Disrupting this amphipathic helix (including the anchoring tyrosine residue) results in loss of the AcrB activity (Liu and Zhang 2017). Similar functional roles of amphipathic helices have been proposed earlier for GPCR activation (Zhang* et al*. 2014), conformational switching of MFS transporters (Jiang* et al*. 2013), and sensing of membrane tension in mechanosensitive channels (Zhang* et al*. 2016). Moreover, the loosely packed TM layer in the AcrB trimer (Fig. 2B) allows the three TM domains tilt independently in response of their individual protonation status. In the AcrB trimer, a differentially phased tilting process of the TM domains is likely to correlate with the packing rearrangement inside the porter layer.

In the high-resolution structures of asymmetric AcrB trimer (*e.g*., 4DX5), the TMs 1–4 in N_TM_ form a groove facing the lipid bilayer. A detergent molecule was found to bind in this groove, suggesting that this site is a ligand- or inhibitor-binding site that may potentially interfere with the coupling between the TM domain and PN1. In the same crystal structure, a similar ligand binding site was also observed in the symmetric position in C_TM_. Interestingly, in a recently reported crystal structure of human cholesterol transporter, NPC1 (PDB ID: 5I31, a member of the RND superfamily), a sterol-sensing domain (SSD) is formed by helices TMs 3–5 (equivalent to TMs 2–4 in AcrB). Both genetic and structural analyses suggest that this region is important for NPC1 function. The head group of the cholesterol molecule bound to the SSD may interfere with the putative signaling β-sheet between the TM domain and the exo-membrane domain. Therefore, the signaling motifs that we proposed here for Ec-AcrB may indeed be a general feature for RND transporters.

We would like to further emphasize that the putative protonation-induced movement requires the presence of a cross-membrane electrochemical potential of protons, particularly the negative-inside ∆*Ψ*. Such precondition was not taken into account in most *in vitro* experiments reported so far, neither for AcrB functional nor for structural studies. Even in molecular dynamic simulations, ∆*Ψ* was usually not included as part of the environmental condition (Schulz* et al*. 2015). The absence of membrane potential may explain why the observed transport rate of AcrB was slow (approx. one substrate molecule per minute) in a proteoliposome-based transport assay (Nikaido and Takatsuka 2009), in contrast to a much higher efficiency during *in vivo* transport (*e.g*., ~100/s for substrate ampicillin) (Li* et al*. 2015).

## Free-Energy Landscape

**Fig. 4:**
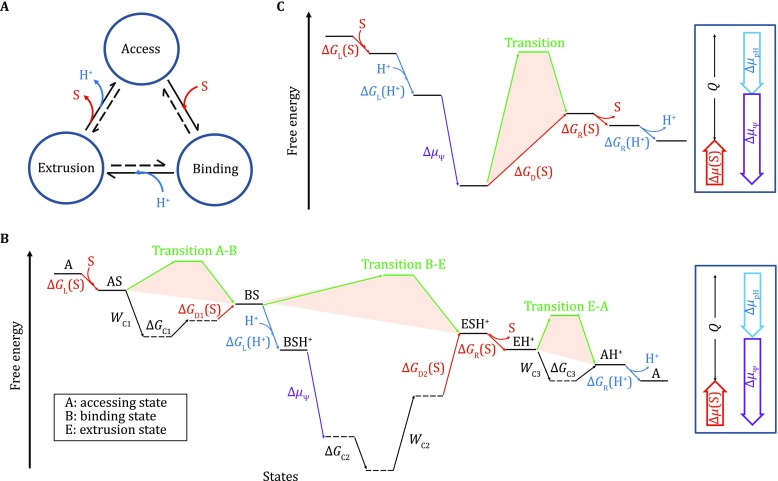
Free-energy landscape of the AcrB protomer. **A** King–Altman plot of the three-state functional cycle of AcrB. The dominant directions of the reactions are shown in solid half arrows. **B** Energy landscape of the AcrB protomer. The plot must satisfy the First and Second Laws of thermodynamics (right-side Box). The vertical axis represents the Gibbs free energy. Horizontal lines represent states, and the horizontal axis is essentially an expansion of the King–Altman plot. Thus, tilted lines represent transitions between states. Transitions associated with the proton binding are indicated in blue, those with the substrate in red, and those with ΔΨ in purple. For simplicity, the electrostatic energy is shown as one package, although it could be separated into two parts associated with proton loading and releasing. In addition, kinetic terms are shown in green. Subscripts “L”, “R”, and “D” stand for energy terms associated with loading, releasing, and differential binding, respectively. The intrinsic conformational energy terms, ∆*G*_C1/2/3_, and energy terms for cooperativity work, *W*_C1/2/3_, are discussed in the main text. In principle, since the transport process cycles, the choice of the starting point is arbitrary. Therefore, the starting and ending states are considered identical, only differing by the amount of heat (*Q*) dissipated during one transport cycle. Thus, the end state must be located below the starting state. Neighboring states may be tightly coupled energetically. In such a case, their sequential order would be arbitrary. Locally, any transition of positive *G* is likely to be driven by a coupled transition of a negative *G* (also see Box 1). **C** Energy landscape plot of the AcrB trimer. Because of cooperativity between the three protomers, the intrinsic conformational energy terms, ∆*G*_C1/2/3_, and energy terms for cooperativity work, *W*_C1/2/3_, shown in **B** cancel each other out. Thus, the plot is simplified. Only one-third of the functional cycle is shown, and the remaining two-thirds are its precise repeats

Functional separation of substrate efflux and proton influx in distinct domains is a characteristic feature of AcrB-like RND transporters (Yamaguchi* et al*. 2015). Because of this separation, Jardetzky’s classical alternating access model (Jardetzky 1966) appears to be not directly applicable to RND transporters. However, the AcrB-like transporters maintain an important feature of most transporters,* i.e*., switching back and forth between conformations of high and low affinities towards a given substrate. In this sense, the two structural units of the AcrB porter domain, namely PN1-PC2 and PN2-PC1, may be analogous to the N- and C-transmembrane domain in an MFS transporter. Whereas the relative movement between the two domains in MFS results in an in-membrane conformational change in agreement to the Jardetzky model, the relative movement between PN1-PC2 and PN2-PC1 results in a conceptually similar conformation alternation, though in the periplasm. A major difference between these two types of transporters is the energy coupling mechanism utilized. For instance, in the case of an MFS antiporter, a direct competition between the substrate and the driving substance, proton or Na^+^, is postulated (Zhang* et al*. 2015). In contrast, the energy coupling mechanism appears to be more complicated in the case of AcrB-like transporters (Eicher* et al*. 2014). As a tool to dissect the energy coupling mechanism of AcrB, we introduce its free-energy landscape plot, as shown in Fig. 4.

By definition, substrates of AcrB are compounds that are able (1) to bind to the access state and (2) to induce subsequent transitions. These rather general criteria allow AcrB to accommodate a broad substrate specificity,* e.g*., that observed in multidrug resistance (Yamaguchi* et al*. 2015). As discussed earlier, the second criterion is equivalent to the ability of the substrate to induce a rotation of the PC1 subdomain, thus triggering protonation in the TM domain. However, for a substrate to meet the first requirement, the loading-site dissociation constant *K*_d,L_ should be smaller than the releasing-site dissociation constant *K*_d,R_, so that the substrate can be captured from a low-concentration environment and be released into a high-concentration environment. Such a difference in substrate binding affinity between two conformations of a transporter is associated with the free-energy term, previously named differential binding energy,* i.e*., ∆*G*_D_ (≡ *RT·*ln(*K*_d,R_/*K*_d,L_) > 0) (Zhang* et al*. 2015) (see Box 1). Without an external energy input, the transporter would not proceed sustainably to transform from a high-affinity state into a lower-affinity state. In order for the functional cycle of substrate export to proceed, the positive ∆*G*_D_ must be compensated by the driving energy,* e.g*., PMF. In the three-step functional cycle, ∆*G*_D_ for the substrate may be further divided into two terms associated with the A-B and B-E transitions (Fig. 4B). Furthermore, a good substrate usually reduces the height of the energy barrier of the transition state(s) (indicated as green horizontal lines in Fig. 4B), but poor substrates or inhibitors lack this ability. Such a reduction can be achieved through stronger binding of the substrate to the transporter at the transition state relative to the “ground” state. This mechanism of reducing the transition-state barrier is likely to also contribute to the substrate specificity observed for transporters (Zhang and Han 2016).

**Box 1:** Definition of the electrochemical potential terms

(i) Free-energy terms of the substrate
$$ \begin{aligned} \Delta \mu \left( {\text{S}} \right) &\equiv {-}RT\cdot{ \ln }\left( {\left[ {\text{S}} \right]_{\text{L}} /\left[ {\text{S}} \right]_{\text{R}} } \right) \\ &= \, \Delta G_{\text{L}} \left( {\text{S}} \right) \, + \Delta G_{\text{D1}} \left( {\text{S}} \right) \, + \Delta G_{\text{D2}} \left( {\text{S}} \right) \, + \Delta G_{\text{R}} \left( {\text{S}} \right) \\ \Delta G_{\text{L}} \left( {\text{S}} \right) &= \, {-}RT\cdot{ \ln }\left( {\left[ {\text{S}} \right]_{\text{L}} /K_{\text{d1}} } \right) \\ \Delta G_{\text{D1}} \left( {\text{S}} \right) &= \, {-}RT\cdot{ \ln }\left( {K_{\text{d1}} /K_{\text{d2}} } \right) \\ \Delta G_{\text{D2}} \left( {\text{S}} \right) &= \, {-}RT\cdot{ \ln }\left( {K_{\text{d2}} /K_{\text{d3}} } \right) \\ \Delta G_{\text{R}} \left( {\text{S}} \right) \\ &= \, {-}RT\cdot{ \ln }\left( {K_{\text{d3}} /\left[ {\text{S}} \right]_{\text{R}} } \right)\end{aligned} $$
(ii) Chemical potential of protons
$$ \begin{aligned} \Delta \mu_{\text{pH}} & \equiv 2. 3\cdot RT\Delta {\text{pH}} \\ & = \Delta G_{\text{L}} \left( {{\text{H}}^{ + } } \right) \, + \Delta G_{\text{R}} \left( {{\text{H}}^{ + } } \right) \\ \Delta G_{\text{L}} \left( {{\text{H}}^{ + } } \right) & = 2. 3\cdot RT \cdot \left( {{\text{pH}}_{\text{L}} {-}{\text{p}}K_{\text{a}} } \right) \\ \Delta G_{\text{R}} \left( {{\text{H}}^{ + } } \right) & = 2. 3\cdot RT \cdot \left( {{\text{p}}K_{\text{a}} {-}{\text{pH}}_{\text{R}} } \right) \\ \end{aligned} $$

The three transitions between successive phases are associated with conformational energy changes, termed ∆*G*_C1_, ∆*G*_C2_, and ∆*G*_C3_, which are intrinsic property of the transporter and are independent of the substrate binding. As a hypothetical example, as shown in Fig. 4B, the B (binding) state is of higher conformational energy than the A (access) state (∆*G*_C1_ > 0). The positive ∆*G*_C1_ suggests that in the absence of external energy, the A-B transition is thermodynamically unfavorable. There are many factors that may contribute to these differential conformational energy terms. For instance, the abovementioned hydrophobic mismatch may be considered as a contributor of the conformational energy in the extrusion phase, thus affecting both ∆*G*_C2_ and ∆*G*_C3_. Since the promoter must return to its initial state after a complete functional cycle, the sum of these energy terms associated with the three conformational changes should be zero:

$$ \Delta G_{\text{C1}} + \Delta G_{\text{C2}} + \Delta G_{\text{C3}} = 0. $$

Conceptually, if the protomers were isolated from the trimer, such conformational energy terms could be determined by measuring its population distribution in different states at equilibrium. Due to the functional coupling between the protomers, however, these energy terms (∆G_C1_, ∆*G*_C2_, and ∆*G*_C3_) may not be determined experimentally for an AcrB-like transporter.

One characteristic feature of the AcrB trimer is the absolute requirement of functional cooperation between the three protomers (Eicher* et al*. 2014). It has been shown that, in a genetically fused AcrB trimer, deactivating one single protomer abolishes the activity of the entire trimer complex (Takatsuka and Nikaido 2009). Furthermore, not all external energy (*i.e*., ∆*µ*_pH_ +*F*∆*Ψ*, where *F* is the Faraday constant and ∆*µ*_pH_ is explained in Box 1) is released as one package to drive the B-E transition. Part of the input energy (*W*_C2_) may be considered as output work for “cooperativity.” In other words, input energy generated by proton influx in one protomer is partially used to allow the other two protomers to overcome their transition barriers as well as thermodynamically unfavorable ∆*G*_C_ terms. Like ∆*G*_C1/2/3_ terms, the energy terms associated with cooperativity is also in balance:

$$ W_{\text{C1}} + W_{\text{C2}} + W_{\text{C3}} = 0. $$

For a hypothetical, isolated, monomeric transporter, relative free-energy levels (and the associated rate constants) of each state together would determine the population probability among different states. This distribution in turn would affect the overall transport rate of the transporter (Hill 1989). The rates of three transitions, A-B, B-E, and E-A, would be different. However, because of the cooperativity within one AcrB trimer, each protomer is present in one of the three major states. Therefore, the population probability of each major state is virtually 1/3. This imposes a strong restriction both on the free-energy landscape and on the kinetics of the transitions between successive states (see Box 2). By automatically adjusting energy terms of cooperative work (*W*_C1_, *W*_C2_, and *W*_C3_), rates at the three transitions are maintained to be the same. For instance, if the substrate concentration at the loading site increases, the pseudo first-order rate constant ([S]_L_ *k*_on_^0^) will increase (Hill 1989). This increased rate constant would shift the population probability from the empty access state (A) to the substrate-bound access state (AS) and would make the A-B transition run faster. Thus, the external energy required for this transition decreases (*i.e*., *W*_C1_ becomes less negative) in order to maintain all three transitions at the same rate. In contrast, in the absence of any substrate at the loading site, the A-B transition as well as the functional cycle stops. In short, the input PMF energy will be distributed dynamically among the three cooperative promoters of the transporter trimer, depending on the types and concentrations of substrate molecules associated with each protomer. For a given type of substrate at a fixed concentration gradient, the cooperativity works *W*_C1_, *W*_C2_, and *W*_C3_ from all three protomers cancel each other out at any given moment. Furthermore, according to the Arrhenius theorem, the pseudo first-order rate constant of a cooperative transporter complex will change by a factor of exp(−*W*_C*i*_/*RT*) (where *i* = 1, 2, 3), relative to the hypothetical rate of an isolated monomeric transporter (Box 2). In addition, ∆*G*_C1_, ∆*G*_C2_, and ∆*G*_C3_ from the three protomers balance each other, albeit they are intrinsic properties of the transporter. Thus, the energy landscape plot of a cooperative AcrB trimer can be presented in a simplified manner, as shown in Fig. 4C. A key feature of this simplified plot is that PMF, including energy terms associated with both ∆pH and ∆*Ψ*, remains to be utilized to compensate the positive ∆*G*_D_(S) in thermodynamic terms and to overcome the transition state energy barrier in kinetic terms.

**Box 2:** Cooperativity

Assumption: The cooperative complex contains* N* copies of identical protomers, and each of them is in a distinct transition-state (or phase) of the functional cycle
(i) Energy conservation
$$ \mathop \sum \limits_{i}^{N} W_{{{\text{C}}i}} = 0 $$
where *W*_C*i*_ is the cooperative work that is output by the protomer in the *i*-th step. A negative *W*_C*i*_ would indicate that the *i*-th step absorbs energy from the cooperative complex
(ii) Arrhenius theorem
$$ k_{i} {\text{e}}^{{ - W{\text{c}}i/RT}} = k_{j} {\text{e}}^{{ - W{\text{c}}j/RT}} $$
where *k*_*i*_ is the (pseudo) first-order rate constant of the *i*-th step in a hypothetical non-cooperative protomer
(iii) Cooperative work
$$ W_{Ci} = \frac{ - RT}{N}{ \ln }\frac{{\mathop \prod \nolimits_{j}^{N} k_{j} }}{{k_{i}^{N} }} = RT{ \ln }\frac{{k_{i} }}{{k_{\text{c}} }} $$
$$ k_{\text{c}} = \left( {\mathop \prod \limits_{j}^{N} k_{j} } \right)^{{\frac{1}{N}}} $$
where *k*_c_ is the pseudo first-order rate constant of the cooperative complex. For instance, for the AcrB trimer, *k*_C_ = (*k*_1·_ *k*_2·_ *k*_3_)^1/3^

## Perspective

The United Nations General Assembly recently unanimously approved a declaration committing nations to a more active battle against threat of microbial resistance to existing drugs, which is one of the most severe and rapidly growing threats to contemporary public health. Massive intergovernmental efforts will be required to achieve such an ambitious undertaking. Since bacteria develop drug resistance naturally in an environment containing antibiotics, studies on the mechanism of multidrug resistance, including those of AcrB-like transporters, are an essential part of our fight against multidrug resistance (Li* et al*. 2015). Based on the progress in structural and functional studies on AcrB-like transporters in the last 15 years, a new energy coupling mechanism is proposed in the current review for this major type of multidrug-resistance transporters. If verified, this mechanism is expected to permit us to de-couple between their substrate binding and energy converting. Experimental approaches stemmed from this mechanistic hypothesis may thus open new avenues for developing novel inhibitors to attack the multidrug resistance mediated by AcrB-like transporters.

## Electronic Supplementary Material

Below is the link to the electronic supplementary material.

Supplementary material 1 (PDF 722 kb)

## Abbreviations

PMF: Proton motive force
RND: Resistance-nodulation-cell division (transporter)
TM: Transmembrane (helix)
