# Supplementary material for: Energy coupling mechanisms of AcrB-like RND transporters
Source: Biophys Rep. 2017 Sep 25;3(4):73–84. doi: 10.1007/s41048-017-0042-y (PMC5719797; doi:10.1007/s41048-017-0042-y)
Supplement: Supplementary file 1 — Supplementary material 1 (PDF 722 kb) [file 41048_2017_42_MOESM1_ESM.pdf]

Supplementary Material

A

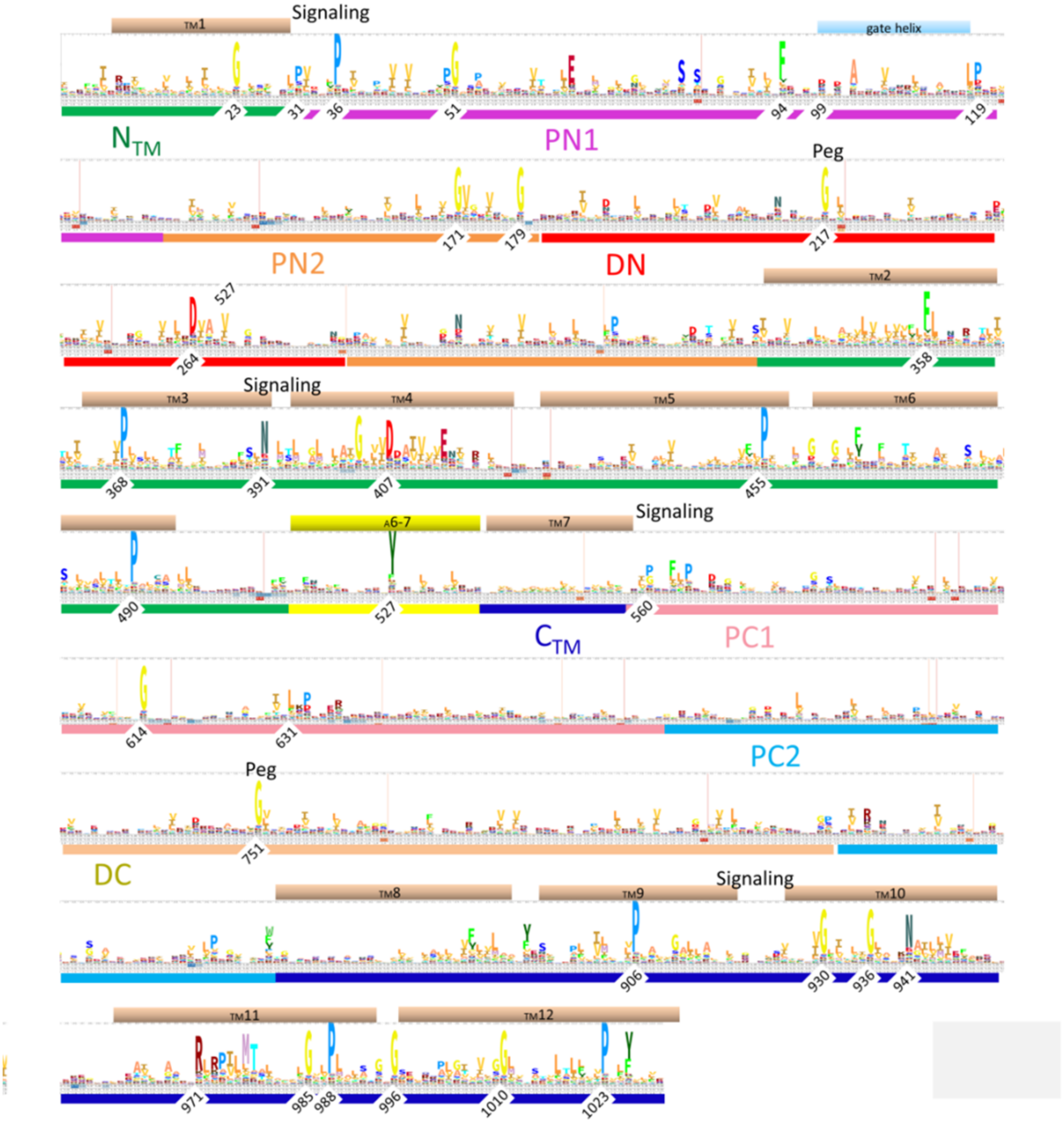

B

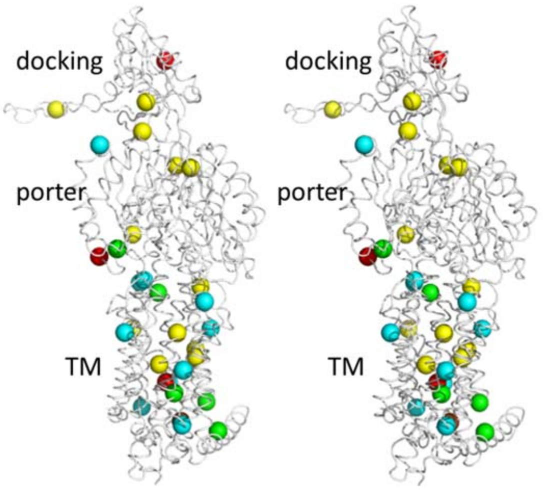

Fig. S1 Conserved residues in AcrB-like transporters. **A** "Weblogo" diagram of AcrB-like transporters, generated by the PFAM website (Finn *et al*. 2008). Total 11,566 amino acid sequences (with an average sequence identity of 24%) were used in generating the original weblogo figure, which was further manually modified and decorated. Selected secondary structures, including signaling motifs (labelled as "Signaling"), are shown on the top. Selected residue numbers from Ec-AcrB are labeled at the bottom. The underline color bars indicate the range of each subdomain, with the color code consistent with that of Fig. 2. **B** Stereo view of the most conserved (*i.e.* nearly irreplaceable) residues in the AcrB protomer. Color code (similar to **A**): Gly, yellow; Pro, cyan; acidic residues, red; basic residues, brown; and others, green
